# Atherosclerosis originating from childhood: Specific features

**DOI:** 10.7555/JBR.37.20230198

**Published:** 2024-05-22

**Authors:** Anastasia V. Poznyak, Alexey A. Yakovlev, Mikhail А. Popov, Elena B. Zhigmitova, Vasily N. Sukhorukov, Alexander N. Orekhov

**Affiliations:** 1 Institute for Atherosclerosis Research, Moscow 121609, Russia; 2 Federal Research and Clinical Center of Intensive Care Medicine and Rehabilitology, Moscow 109240, Russia; 3 Department of Cardiac Surgery, Moscow Regional Research and Clinical Institute, Moscow 129110, Russia; 4 Laboratory of Angiopathology, Institute of General Pathology and Pathophysiology, Moscow 125315, Russia

**Keywords:** atherosclerosis, childhood, pediatrics, cardiovascular disease

## Abstract

Atherosclerosis is extremely widespread. Traditionally, it is considered a disease of older people, who most often experience problems with the heart and blood vessels. While much attention from the scientific community has been paid to studying the association between aging and atherosclerosis, as well as its consequences, there is evidence that atherosclerosis occurs at an early age. Atherosclerosis may form both during intrauterine development and in childhood. Nutrition plays an important role in childhood atherosclerosis, along with previous infectious diseases and excess weight of both the child and the mother. In the present review, we examined the development of atherosclerosis and the prerequisites in childhood.

## Introduction

Atherosclerosis was once considered a localized disease characterized by cholesterol accumulation with arterial stenosis that limits blood flow. However, the definition of the origin and development of atherosclerosis has changed significantly in recent decades. Today, it has become clear that atherosclerosis is a chronic inflammatory process of the arterial wall, resulting from a combination of genetic changes, an imbalance of lipid metabolism, and a maladaptive immune response^[1]^.

Cardiovascular diseases primarily caused by atherosclerosis, such as stroke or ischemic heart disease, typically manifest in adulthood, but there is convincing evidence indicating that vascular changes associated with atherosclerosis begin in childhood. These changes become diagnosable in later stages during adulthood^[2]^. Common risk factors for cardiovascular diseases include diabetes, hypercholesterolemia, smoking, obesity, and hypertension. Surprisingly, about 20% of cardiovascular events occur without the manifestation of the above-mentioned risk factors^[3]^.

The phenomenon of early atherosclerosis first gained attention through autopsy studies conducted during the Korean and Vietnam Wars, and these studies revealed that a significant proportion of young soldiers, averaging 22 years old, showed evidence of atherosclerosis in their coronary arteries^[4–5]^. The subsequent investigation focused on young American children involved in fatal motor accidents, demonstrating a high prevalence of early-stage atherosclerosis, and over 50% of children aged 10 to 14 displayed lipid-laden macrophages in their arteries^[6]^.

In Japan, a nationwide autopsy-based study covering individuals ranging from one month to 39 years old showed fatty streaks in 29% of aortas in children younger than one-year-old and in 3.1% of coronary arteries in children aged one to nine years old^[7]^. A follow-up examination conducted 13 years later showed an increase in the prevalence and extent of coronary artery lesions among autopsied subjects who passed away in their third and fourth decades of life^[8]^. The Bogalusa Heart Study evaluated 204 young patients aged two to 39 years, assessing the presence and extent of fatty streaks and fibrous plaques in the aorta and coronary arteries, and found that the prevalence of fatty streaks and fibrous plaque lesions increased with age^[9]^.

Therefore, the present review aims to provide a comprehensive summary of atherosclerotic features specific to the pediatric population. The first part of our manuscript delves into the mechanistic aspects, specifically examining the role of lipoproteins and abnormalities in their metabolism. The second part is dedicated to exploring the particular risk factors that manifest during childhood. Additionally, we have devoted attention to fetal studies.

## Early lesion formation

The primary risk factors associated with arterial damage at an early age include those contributing to progressive lesions that cause coronary artery diseases in adulthood. All of these factors contribute to more advanced atherosclerotic lesions and atherosclerotic plaques^[10]^. These factors include systolic and diastolic blood pressure, the elevated body mass index, smoking, diabetes mellitus, the increased levels of low-density lipoprotein (LDL) cholesterol, and low levels of high-density lipoprotein (HDL) cholesterol. Autopsy data demonstrated that, as these risk factors increased, the severity of the asymptomatic cardiovascular disease increased in individuals aged two to 39 years^[11]^.

The earliest precursors of lesions are fatty streaks, which are present from early childhood and manifest by the age of 20 or 30^[12]^. Between 15 and 34 years old, these lesions become raised, and plaques spread and expand quickly. Adolescents and young adults were found to have relatively high levels of atherosclerosis, including fibrous plaques^[13]^. In vulnerable anatomical places, fatty streaks progress to the raised lesions, and the primary sites for the appearance of fatty streaks are vascular surfaces exposed to turbulent flow, which are also the same areas prone to neglected lesions that lead to thrombosis and plaque rupture^[14]^.

Observational studies of the autopsies have provided valuable data on the severity and timing of atherosclerotic lesions^[15–16]^. Noninvasive studies have demonstrated both functional and anatomical changes in atherosclerosis among young individuals. As a result, the identified risk factors collectively predict the wall thickness of the internal carotid artery in young people, which progresses with age^[17–18]^.

Based on the obtained epidemiological and observational data, the following conclusions were drawn: (1) atherosclerosis manifests itself at an early age; (2) the presence and degree of risk factors are strongly associated with the severity of atherosclerosis; (3) the cumulative impact of several risk factors is exponentially greater than individual factors; and (4) with age and an increase in the number of risk factors, the severity of atherosclerosis significantly increases.

## The role of LDL

An elevated LDL cholesterol level is a significant risk factor for atherosclerosis and cardiovascular diseases, emphasizing the importance of maintaining low LDL levels from childhood to prevent heart-related issues in adulthood^[19]^. The desialylation of LDL leads to the formation of an electronegative subfraction that is rich in desialylated LDL particles, which have smaller sizes, altered compositions, and increased proatherogenic properties, highlighting the significance of desialylated LDL in the development of atherosclerosis and its potential use as a target for quantification and isolation in the blood^[20]^. Moreover, the desialylation of LDL exposes the galactose residue, which binds to lectins and may be quantified and isolated using lectin-based assays, and the resulting desialylated LDL is highly proatherogenic and may lead to increased cholesterol deposits in cells^[21]^. Furthermore, the increased levels of desialylated LDL, particularly in atherosclerotic patients, are associated with higher atherogenicity and susceptibility to oxidation, indicating the potential role as a biomarker and target for understanding and managing cardiovascular diseases^[22]^.

The advanced desialylation of LDL is also considered an early step in atherogenic lipid modification. In addition to serum sialidase, which plays a key role in enzymatically removing sialic acid from LDL, oxidative stress associated with atherosclerosis also leads to a non-enzymatic desialylation, resulting in the desialylated LDL with the reduced antioxidants, an accelerated degradation, and a modified apoB, increasing its proatherogenic potential^[23]^. Desialylated LDL not only impairs reverse cholesterol transport but also inhibits cholesterol-esterifying activity, while inflammation up-regulates galactose-specific lectins in macrophages, promoting the uptake of desialylated LDL^[24]^.

The desialylated LDL subfraction triggers the production of proatherogenic IgG auto-antibodies, leading to an increased uptake of LDL by aortic cells and potentially contributing to atherogenesis^[25]^. Furthermore, the presence of anti-LDL antibodies in the bloodstream results in the formation of cholesterol-containing circulating immune complexes with atherogenic properties, emphasizing the significant proatherogenic potential of these antibodies; and the characteristics of LDL extracted from immune complexes are similar to desialylated LDL, including a smaller size, a higher density and electronegative charge, a reduced sialic acid content, the elevated levels of oxysterols, and a comparable lipid peroxide content^[26]^.

Thus, the desialylation of LDL plays a crucial role in the development of atherosclerosis and cardiovascular diseases. Understanding and managing desialylated LDL may provide valuable insights into the management of cardiovascular diseases.

### Lipoprotein (a) [Lp(a)]

The study by Raitakari *et al*^[27–28]^ has suggested that the measurement of Lp(a) should be included in the assessment of cardiovascular risk, because it has been shown to independently contribute to the risk of atherosclerotic cardiovascular diseases in both children and adults, in addition to LDL cholesterol. Children with elevated levels of both Lp(a) and LDL cholesterol have a significantly higher risk of premature atherosclerotic cardiovascular diseases^[29]^. Screening for Lp(a) during childhood may identify a stable and genetically determined risk factor that aids in earlier risk stratification and the management of other cardiovascular risk factors^[30]^.

## Intimal structure and thickening

The arterial intima, which is the inner layer of arteries, thickens with age and consists of fibrocellular tissue^[31]^. Two types of intimal thickening have been identified: eccentric intimal thickening and diffuse intimal thickening (DIT)^[32]^. DIT is considered a normal occurrence in human arteries, exhibiting a well-organized structure comprising smooth muscle cells (SMCs), elastin, and proteoglycans. DIT is often associated with atherosclerosis and is mainly found in atherosclerosis-prone arteries^[33]^.

Interestingly, DIT has been detected even in fetuses and infants, with a gradual increase in the intima-to-media ratio as they grow older^[34]^. This thickening is present in the proximal left anterior descending artery both before and after birth. The formation of DIT is believed to be a physiological adaptation to mechanical stresses^[35]^. The thickness of DIT is influenced by factors such as blood flow grade and shear stress, with hypertensive arteries showing a greater thickness^[36]^. However, the association between shear stress and intimal thickness is not consistent across all arteries^[37]^. Further investigation is needed to fully understand the mechanisms underlying DIT formation and its impact on the health of children and fetuses.

### Intima in embryonic arteries

During the embryonic stage, human aorta contains SMCs that vary in their ultrastructure, shape, packing, and surface microrelief. A previous study demonstrated the heterogeneity of intimal cell populations in the adult human aortas, found that elongated and stellate SMCs were present in both embryonic and adult aortas, and revealed that the foundation for this phenomenon was established during embryogenesis between 18 and 28 weeks^[38]^.

The cells in different layers of the embryonic aortic media exhibit diverse shapes and form various contacts with each other. Notably, SMCs are in the closest proximity to the lumen and may penetrate the intima without contacting other cells. This observation may be linked to the formation of the aortic intima or signify the migration of cells from the intima to the media. In human aorta, intimal cells appear during the early prenatal period, but their nature and origin remain unexplored and are directly connected to medial SMCs through the internal elastic membrane^[39]^. SMCs may emerge in the intima because of cell migration from the media. Another study investigated the embryonic intima and found the presence of isolated or clustered cells, suggesting that a single cell from the media may migrate to the intima, undergo division, and form a cluster; the same study also demonstrated that all subendothelial cells in the embryonic aorta contain SMC antigens; however, despite these findings, two predominant cell populations consisting of spherical and elongated cells were isolated from early stages of embryogenesis in human aortas^[40]^. Ultrastructurally, spherical cells exhibited a poor differentiation, whereas the elongated cells displayed key characteristics of the differentiated SMCs. These cell populations differ in terms of cell packing, with spherical cells densely packed with minimal connective tissue between cells, while the elongated cells are separated by distinct layers of connective tissue^[41]^. The aortas of 18 to 28 weeks old embryos showed two cell types: poorly differentiated stellate cells near the endothelium and spindle-shaped SMCs in the medial cell population, but intimal-like cells with processes were also found in the innermost part of the media^[42]^. The presences of spheroid cells in early embryos, cells with processes in later embryos, and subendothelial stellate cells in the adult aorta suggest that spheroid cells may be precursor cells for later-developing cells and stellate cells during embryogenesis^[42]^.

## Fetal studies

**Figure 1:**
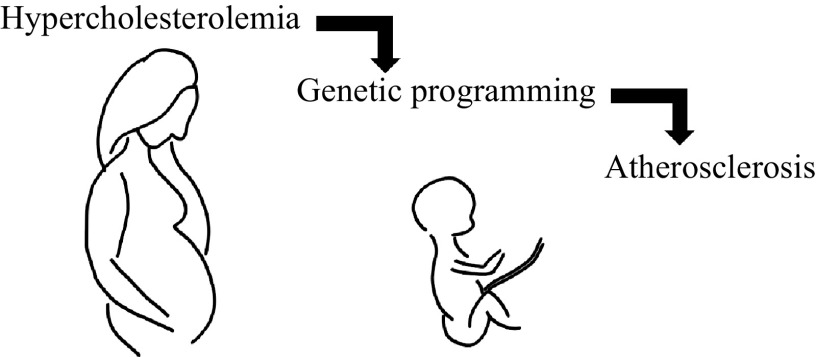
The potential association between maternal hypercholesterolemia and child atherosclerosis.

Maternal hypercholesterolemia increases fetal fatty streak formation, potentially harming the unborn child's health^[43]^. Maternal-fetal cholesterol correlation impacts lesion formation, with an accelerated atherosclerosis in the affected children. Antioxidants and cholesterol-lowering drugs mitigate atherosclerosis risk during fetal development and postpartum^[44]^. Animal models highlight gene expression differences, linking fetal damage and genetic programming to postnatal atherogenesis, and ***Fig. 1*** illustrates this association. Cholesterol-lowering and antioxidant treatments positively influence intrauterine programming, reducing postpartum atherogenesis susceptibility^[45–46]^.

## Infections in childhood as a predisposing factor for atherosclerosis development

It is known that an incorrect lifestyle increases the risk of developing atherosclerosis. Additionally, it has also been proven for a long time that changes associated with atherosclerosis are manifested even at the early stages of development. For example, vascular intima thickening is observed in children, and fatty streaks appear at a young age^[47–48]^. Recent studies indicate that the basis of atherosclerosis is laid in early childhood, regardless of lifestyle^[47–49]^. Scientists have identified coronary intimal thickening in more than 30% of children; regardless of sex, age, and causes of death, proatherogenic changes in at least two arteries were detected in most adolescents^[49]^.

Despite children not being exposed to traditional risk factors, such as smoking, obesity, and diabetes, the cause of artery deformity may be a viral infection. Acute viral infection significantly contributes to an increase in proatherogenic changes in individuals under the age of 15 years, whereas in adults, infections are often associated with an accelerated atherosclerosis progression^[50]^. The systemic cytokine response acts as an indirect catalyst for atherogenesis and progression, and pathogenic microorganisms damage vascular cells, exacerbating the condition. Even after purification, intracellular pathogens may affect the development of atherosclerosis for a long time, because they may bypass the protective mechanisms of the host body^[51–53]^. However, at the same time, the seroepidemiological associations between atherosclerosis and infectious pathogens remain unconfirmed, because the observation results are distorted by confounding factors. In theory, these factors may explain the ineffectiveness of antibacterial therapy and vaccination in the prevention of cardiovascular diseases^[54]^.

An infectious history may establish a foundation susceptible to atherogenesis, even though the history does not solely determine the possibility of developing the disease. It is reported that nutrition and an infectious history during development may influence the possibility of atherosclerosis in adulthood^[2]^. There is a concept suggesting that certain types of infections, if not properly treated in childhood, are the primary causes of the pro-atherosclerotic effects of the metabolic processes in later life^[55]^.

Since 1950, at least 13 studies have been conducted, documenting that infections experienced in childhood may be associated with early manifestations of atherosclerosis. For example, infections could be the cause of the thickening of endothelial vessels and a manifestation of coronary heart disease^[2]^. Notably, 12 of these studies were conducted in the developed Western countries, and one in Indonesia, where the study groups, on average, had a higher body mass index than the national average, indicating that these subpopulations had adapted to Western eating habits. According to the results, three studies found that patients with a history of infectious diseases and overweight had higher prevalence rates of cardiovascular diseases, compared with those with a body mass index of < 25 kg/m^2[56]^.

As a result, we conclude as follows: (1) infections experienced in childhood do not appear to directly cause atherosclerosis; however, they, along with overeating, are associated with the development of atherosclerotic changes; (2) individuals who are often susceptible to infections, but at the same time maintain a low-calorie diet, have a low prevalence of atherosclerosis; and (3) the combinations of eating habits and infections in older age significantly affect the development of atherosclerosis.

## Malnutrition in childhood as a predisposing factor for atherosclerosis

The critical phase of development occurs during fetal and early childhood stages, affecting the endocrine, nervous, and immune systems^[57]^. External factors such as nutrition and infections impact these systems, leading to growth and development issues. Insufficient or excessive nutrition in childhood causes persistent epigenetic changes, modifying cardiovascular health and promoting vascular inflammation^[58]^. A preference for fatty foods results in atherogenic profiles and insulin resistance^[59]^. Maternal body mass index and gestational diabetes predict obesity in offspring, potentially because of early fetal insulin resistance^[58–59]^. The association between maternal malnutrition and low birth weight suggests potential cardiovascular effects, requiring further investigations on carotid intima thickness and childhood hunger^[60]^.

The longitudinal study REACTION (Risk Evaluation of Cancers in Chinese Diabetic Individuals: A Longitudinal Study) explored cardiovascular disease risk in individuals exposed to hunger during development, and the results showed a higher cardiovascular disease risk in those exposed to hunger in childhood/adolescence, compared with prenatal exposure^[61]^. Hunger effects were mediated by metabolic syndrome that leads to insulin resistance, a common phenomenon in both malnutrition and overeating^[61]^. Experimental studies highlight the role of the gut microbiome in diabetes and obesity pathogenesis as well as in the association between eating disorders and atherogenicity^[61]^. Li *et al*^[61–62]^ further validated the hypothesis that exposure to hunger in childhood posed a greater risk of atherosclerosis than the exposure during prenatal development did. If these results are confirmed in the future, they will significantly impact public health^[61–62]^.

## Obesity in childhood as a predisposing factor for atherosclerosis

**Figure 2:**
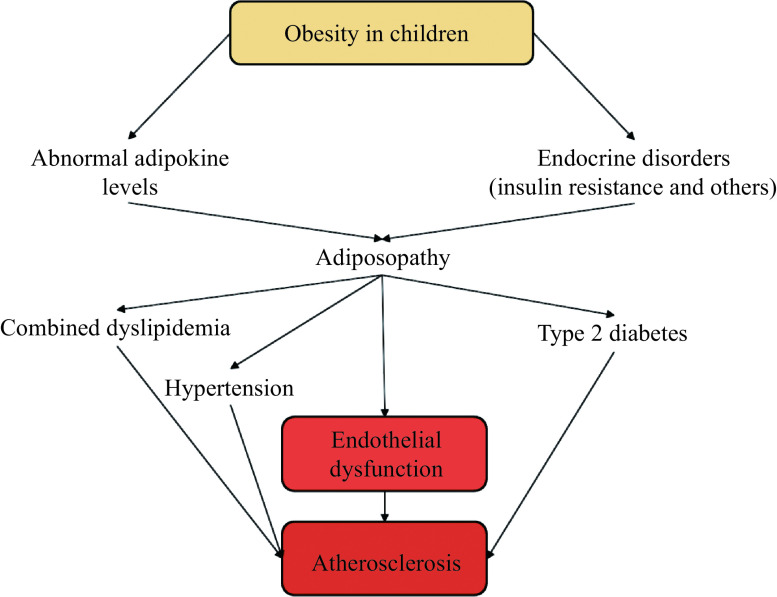
Implication of childhood obesity in atherosclerosis development.

While obesity itself may increase the risk of cardiovascular diseases, the primary risk depends on concurrent cardiometabolic risk factors (***Fig. 2***). Functional and anatomical disruptions in adipocytes and fatty tissues contribute to the pathophysiological process known as adiposopathy, and individuals with the same degree of obesity may have different degrees of cardiometabolic disorders^[63]^. The presence of adiposopathy may be detected with oxidative stress biomarkers, abnormal adipokine levels, and endocrine disorders, such as insulin resistance commonly observed in people with obesity. Such disorders may lead to combined dyslipidemia, characterized by high triglyceride levels, low HDL cholesterol levels, high non-HDL cholesterol levels and enhanced LDL particles, and reduced particle sizes as well as hypertension and type 2 diabetes that are commonly seen in overweight and obese youth^[64]^.

These risk factors, along with fat deposit-associated inflammation and oxidative stress, may lead to endothelial dysfunction and the initiation and development of atherosclerosis. Investigations highlight that while obesity itself increases the risk of cardiovascular diseases, the presence and severity of cardiometabolic disorders vary among individuals with the same degree of obesity, emphasizing the pivotal role of adipocyte dysfunction and adiposopathy in the pathophysiological processes underlying these disorders. These processes include oxidative stress biomarkers, abnormal adipokine levels, and endocrine disturbances, such as insulin resistance. The identification of these risk factors, along with the accompanying inflammation, oxidative stress, and endothelial dysfunction, contributes to a better understanding of the development and progression of atherosclerosis.

## Conclusions and perspectives

In the present review, we addressed various risk factors in children, contributing to the development of atherosclerosis, along with the hallmarks of early atherosclerosis. Studies have shown that the greater the "risk burden", the earlier the development of atherosclerosis is likely to occur, although it stretches over decades. The most severe risk factor in children is malnutrition, where both insufficient and excessive nutrition may, in one way or another, increase the risk of developing atherosclerosis. The most interesting, in our opinion, is fetal studies. It has been observed that in approximately 50% of the cases, fetuses of mothers suffering from hypercholesterolemia have a significant increase in the formation of fatty streaks in the arteries. Therefore, maternal cholesterol levels may influence fetal cholesterol levels, thereby increasing the risk of developing related diseases.
